# Cerebro-Spinal Meningitis

**Published:** 1865-12

**Authors:** C. A. White


CHICAGO

### MEDICAL JOURNAL.

VOL. XXII.] DECEMBER, 1865. [No. 12

### ORIGINAL COMMUNICATIONS.

### CEREBRO-SPINAL MENINGITIS.

Reported by C. A. WHITE, M. D.

In the night of May 16th, 1865, I was called in haste to see a man aged — years, of the appearance of a good constitution. He had been feeling some depression of the vital powers for some days, and being himself a physician, though not in practice, he had prescribed for himself. He was sitting in his room with a friend at 11½ o'clock, when he arose and went towards his bed, but fell upon his face before he reached it. His friend immediately went to his assistance, and found him in convulsions, and immediately sent for me. I found him insensible, extremities cold, face flushed, abdomen tympanitic and very tender, particularly the epigastric region, pupils unequally dilated, strabismus, head thrown back, nuchal region very tender.

The convulsions and intervals were each of about ten minutes duration. During the convulsions the pulse became very weak and slow, the breathing labored and often suspended from one to two and a-half minutes at a time, the jaws set, hands clenched, and arms and legs extended. During the intervals he would grope for imaginary objects before him, or which seemed to cover his eyes.

The convulsions continued until near 4 o'clock of the morning of the 17th, diminishing in intensity, with longer intervals, the pulse gradually improving in volume and frequency, but somewhat irregular.

As the convulsions passed off, the warmth of surface returned and he fell into profound coma, yet he moaned with pain, and was immediately aroused when the epigastric or nuchal regions were touched. There was evident imperfection of hearing and vision, some soreness of the joints, but no hyperæsthesia of the general surface.

As deglution was impossible I was confined at first to external remedies. I made immediate application of dry heat to the feet, legs and arms, by means of oats heated in pans in the stove and emptied into pillow slips, which being loosely tied allowed of free packing around the limbs. I then applied mustard poultices over the stomach, and to the back of the neck and occiput. When the convulsions ceased, deglutition was possible but very difficult. Articulation was also very difficult. I ordered Tinct. Ferri Chloride ʒ 3 ; Tinc. Cantharidis ʒ 5. Misce, Sig. Take 50 drops every hour.

8 o'clock A. M.—Extremities warm, pulse 65, and a little fuller than natural, tongue of natural dimensions, dark, dry, and fissured in the middle; coma not so deep, pain great as before, passed urine of natural quantity and color. Continued the tinctures, but lessened the dose to 40 drops.

11 o'clock A. M.—Coma passing off; tongue moist but dark; pulse 60 and full; tympanitis somewhat relieved; pain not so intense; less disposition to throw the head back.

3 o'clock P. M.—Patient quite rational; comprehends his case and has given his friend directions concerning his business while he is sick, and if he should die, "gave him commandment concerning his bones," and awaited the result like a man. Tongue moist; pulse 68 and of natural fullness; symptoms of strangury present, from effects of the medicine. Discontinued the tinctures, and gave castor oil with 20 drops turpentine.

5 o'clock P. M.—Patient about the same; difficulty of swallowing nearly gone.

8½ o'clock P. M.—Convulsions returned suddenly while I was with him anticipating such a result, although there was no other admonition than a reduction of the pulse in force and frequency. They were of the same character as the others, but less severe, and with less disposition to throw the head back. Treatment, same as before.

They continued an hour and a-half when he fell into a comatose condition, but aroused in half an hour to have an evacuation from the bowels, which was repeated three times within an hour, with a free passage of urine, the warmth of surface meantime improving.

12½ o'clock A. M. of the 18th.—Coma continues, but pulse improving; deglutition and articulation almost impossible.

7½ o'clock A. M.—Coma somewhat relieved; pulse 58 and full; tongue dry again; deglutition very difficult; moaning with pain. Applied mustard poultices to the neck and epigastric region as before, and also gave the tinctures of iron and cantharides as in the first instance; tympanitis gone, but pain in the nuchal and epigastric regions continued.

10½ o'clock A. M.—Pulse variable, from 56 to 70 within 15 minutes, and slightly irregular; coma slight, surface warm, abdomen tender, but painless; tongue becoming moist.

1 o'clock P. M.—Consciousness complete, with but little disposition to doze; has just passed a quantity of high-colored urine; pain decreasing; pulse 56; tongue moist.

4 o'clock P. M.—Patient comfortable; coma gone; pain and tenderness of the neck much less, but feels a deep-seated acute pain in the region of the foramen magnum whenever he moves his head suddenly; passes urine of natural quantity, and rather high color. Symptoms of strangury becoming apparent, the tinctures were discontinued; pulse 54, not very strong; tongue moist and dark; general surface of natural warmth and moisture.

From 8 till 12 o'clock, pulse 56, of good volume; urine of natural quantity, and rather high color; slept well during the night; gave no medicine.

8 o'clock A. M. of the 19th.—Pulse of good fullness, but falls to 50 while sleeping; ordered Sulph. Quinine et Pulv. Capsici, of each two grains every two hours, also beef tea, with an occasional tablespoonful of hot whisky punch.

From this time until towards morning of the 20th, he remained comfortable, but with considerable prostration, which was relieved by the use of stimulants.

9 o'clock A. M. of the 20th.—Patient comfortable and improving; soreness of neck and stomach diminishing. Discontinued all medicines, and gave beef-tea and egg-nog.

From this time forward the improvement has been steady and comparatively rapid.

I have no doubt whatever of my diagnosis of this case, but the following variations from the typical forms of so-called spotted fever are worthy of remark.

The attack was not attended at its commencement by any distinct rigor, although there was great coldness of the extremities.

No eruption appeared on any part of the body.

The pulse never went above 70, and this only during the intermission.

Thirst was never great during the whole attack.

The delirium was at no time violent.

